# Blood Pressure Sign in Pneumothorax and Pleural Effusion

**Published:** 1918-08

**Authors:** 


**Blood Pressure Sign in Pneumothorax and Pleural Effusion.** Williamson, London Lancet, says that while ordinarily the blood pressure in the arms and legs is nearly the same, distention of the pleura causes a diminution of 10-20 m.m. pressure in the leg as compared with the arm of the same side. At first thought, this seems natural but even the crudest conception of vascular anatomy leads us to inquire as to the explanation and, especially whether, if direct pressure on the aorta acts as a mechanic cause, why the pressure is not equally reduced in both legs. If this sign is corroborated, the explanation must be sought in a rather recondite innervational phenomena, through the vaso-motor nerves.

